# Recommendations for tilt table testing and other provocative cardiovascular autonomic tests in conditions that may cause transient loss of consciousness: Consensus statement of the European Federation of Autonomic Societies (EFAS) endorsed by the American Autonomic Society (AAS) and the European Academy of Neurology (EAN)

**DOI:** 10.1007/s10286-020-00738-6

**Published:** 2021-03-19

**Authors:** Roland D. Thijs, Michele Brignole, Cristian Falup-Pecurariu, Alessandra Fanciulli, Roy Freeman, Pietro Guaraldi, Jens Jordan, Mario Habek, Max Hilz, Anne Pavy-Le Traon, Iva Stankovic, Walter Struhal, Richard Sutton, Gregor Wenning, J. Gert Van Dijk

**Affiliations:** 1 Department of Neurology, Leiden University Medical Centre, Leiden, The Netherlands; 2 Stichting Epilepsie Instellingen Nederland (SEIN), Heemstede, The Netherlands; 3 Faint and Fall Programme, Department of Cardiology, Ospedale San Luca, IRCCS Istituto Auxologico Italiano, Milan, Italy; 4 Department of Cardiology and Arrhythmologic Centre, Ospedali del Tigullio, 16033 Lavagna, Italy; 5 Department of Neurology, County Emergency Clinic Hospital, Transilvania University, Brasov, Romania; 6 Department of Neurology, Medical University of Innsbruck, Innsbruck, Austria; 7 Beth Israel Deaconess Medical Center, Harvard Medical School, Boston, MA USA; 8 IRCCS Istituto Delle Scienze Neurologiche di Bologna, Bologna, Italy; 9 German Aerospace Center (DLR), Institute of Aerospace Medicine, Cologne, Germany; 10 Chair of Aerospace Medicine, University of Cologne, Cologne, Germany; 11 University Hypertension Center, Cologne, Germany; 12 Referral Center for Autonomic Nervous System, Department of Neurology, School of Medicine, University Hospital Center Zagreb, University of Zagreb, Kispaticeva 12, 10000 Zagreb, Croatia; 13 Department of Neurology, University Erlangen-Nuremberg, Erlangen, Germany; 14 Department of Neurology, Icahn School of Medicine at Mount Sinai, New York, NY USA; 15 Neurology Department, French Reference Center for MSA, University Hospital of Toulouse and INSERM U 1048, Toulouse, France; 16 Clinical Center of Serbia, Neurology Clinic, University of Belgrade, Belgrade, Serbia; 17 Department of Neurology, University Clinic Tulln, Karl Landsteiner University of Health Sciences, Tulln, Austria; 18 Department of Cardiology, National Heart and Lung Institute, Hammersmith Hospital, Ducane Road, London, W12 0NN UK

**Keywords:** Transient loss of consciousness, Syncope, Tilt table testing, Vasovagal, Reflex syncope, Orthostatic hypotension, Psychogenic pseudosyncope

## Abstract

An expert committee was formed to reach consensus on the use of tilt table testing (TTT) in the diagnosis of disorders that may cause transient loss of consciousness (TLOC) and to outline when other provocative cardiovascular autonomic tests are needed. While TTT adds to history taking, it cannot be a substitute for it. An abnormal TTT result is most meaningful if the provoked event is recognised by patients or eyewitnesses as similar to spontaneous events. The minimum requirements to perform TTT are a tilt table, a continuous beat-to-beat blood pressure monitor, at least one ECG lead, protocols for the indications stated below and trained staff. This basic equipment lends itself to the performance of (1) additional provocation tests, such as the active standing test, carotid sinus massage and autonomic function tests; (2) additional measurements, such as video, EEG, transcranial Doppler, NIRS, end-tidal CO_2_ or neuro-endocrine tests; and (3) tailor-made provocation procedures in those with a specific and consistent trigger of TLOC. TTT and other provocative cardiovascular autonomic tests are indicated if the initial evaluation does not yield a definite or highly likely diagnosis, but raises a suspicion of (1) reflex syncope, (2) the three forms of orthostatic hypotension (OH), i.e. initial, classic and delayed OH, as well as delayed orthostatic blood pressure recovery, (3) postural orthostatic tachycardia syndrome or (4) psychogenic pseudosyncope. A therapeutic indication for TTT is to teach patients with reflex syncope and OH to recognise hypotensive symptoms and to perform physical counter manoeuvres.

## Introduction

Tilt table testing (TTT), while initially developed to study physiological compensatory responses to orthostatic stress, proved useful as a diagnostic test for vasovagal syncope (VVS) in 1986 [1]. TTT is now widely used in clinical practice with a variety of protocols, variants and extensions. On 6 July 2018, the European Federation of Autonomic Societies (EFAS) organised a round table discussion to obtain consensus on the use of TTT in the diagnosis of disorders that may cause transient loss of consciousness (TLOC) and to outline when other provocative cardiovascular autonomic tests are needed. The 2018 guidelines of the European Society of Cardiology [2] and the 2011 EFAS/American Autonomic Society (AAS) Consensus paper [3] were used as starting points. Definitions of various conditions and tests are used as presented in these sources, where necessary preference was given to the more recent ESC guidelines. Two authors (RDT and JGvD) searched PubMed for additional articles from 2014, with the keywords ‘tilt table’ AND ‘syncope’ OR ‘orthostatic intolerance’ (449 hits). We also cite occasional earlier articles and reviews, when these are relevant. The resulting consensus statement was reviewed by all participants, discussed at a second session on 30 June 2019, a third session on 27 January 2020, and sent for endorsement to the autonomic nervous system (ANS) panel of the European Academy of Neurology (EAN). This consensus statement reflects the opinion of experts in the field, but is not a formal evidence-based clinical guideline.

## Indications

**Fig. 1:**
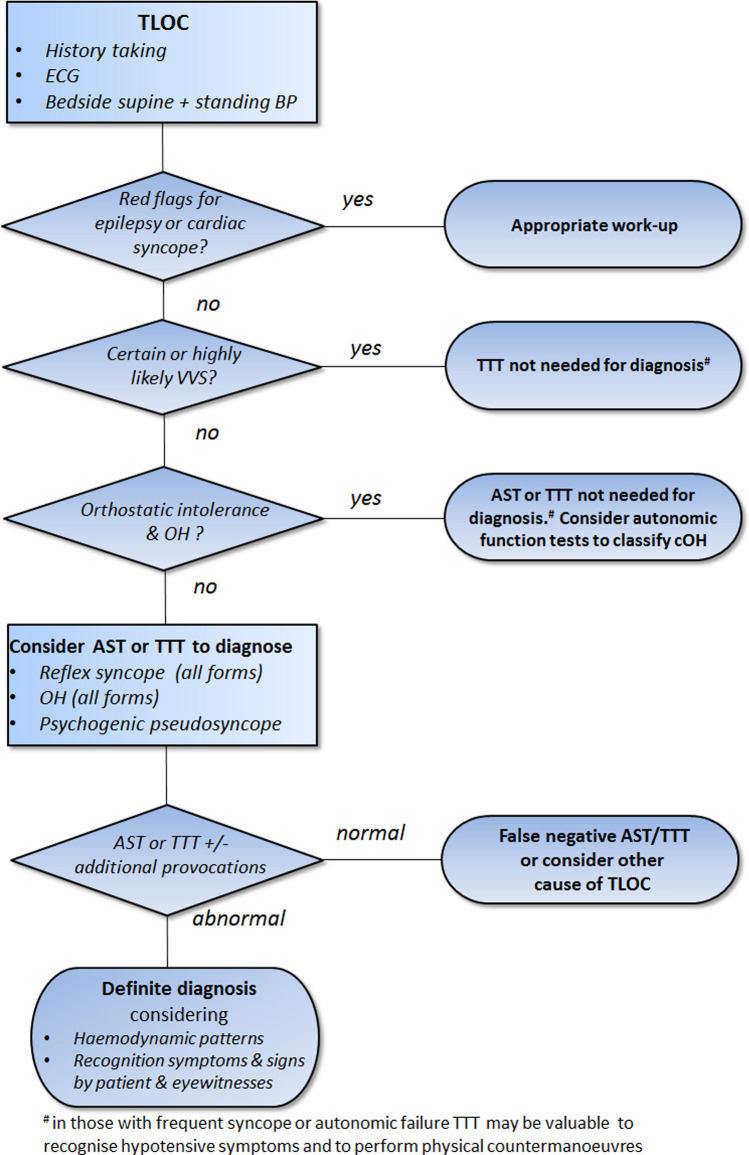
Flow chart of the diagnostic work-up following the initial evaluation of transient loss of consciousness (TLOC), i.e. history taking, ECG and bedside supine and standing blood pressure measurements. *AST* active standing test with continuous blood pressure measurements, *BP* blood pressure, *cOH* classic orthostatic hypotension, *CSM* carotid sinus massage, *TLOC* transient loss of consciousness, *TTT* tilt table testing, *VVS* vasovagal syncope

**Fig. 2:**
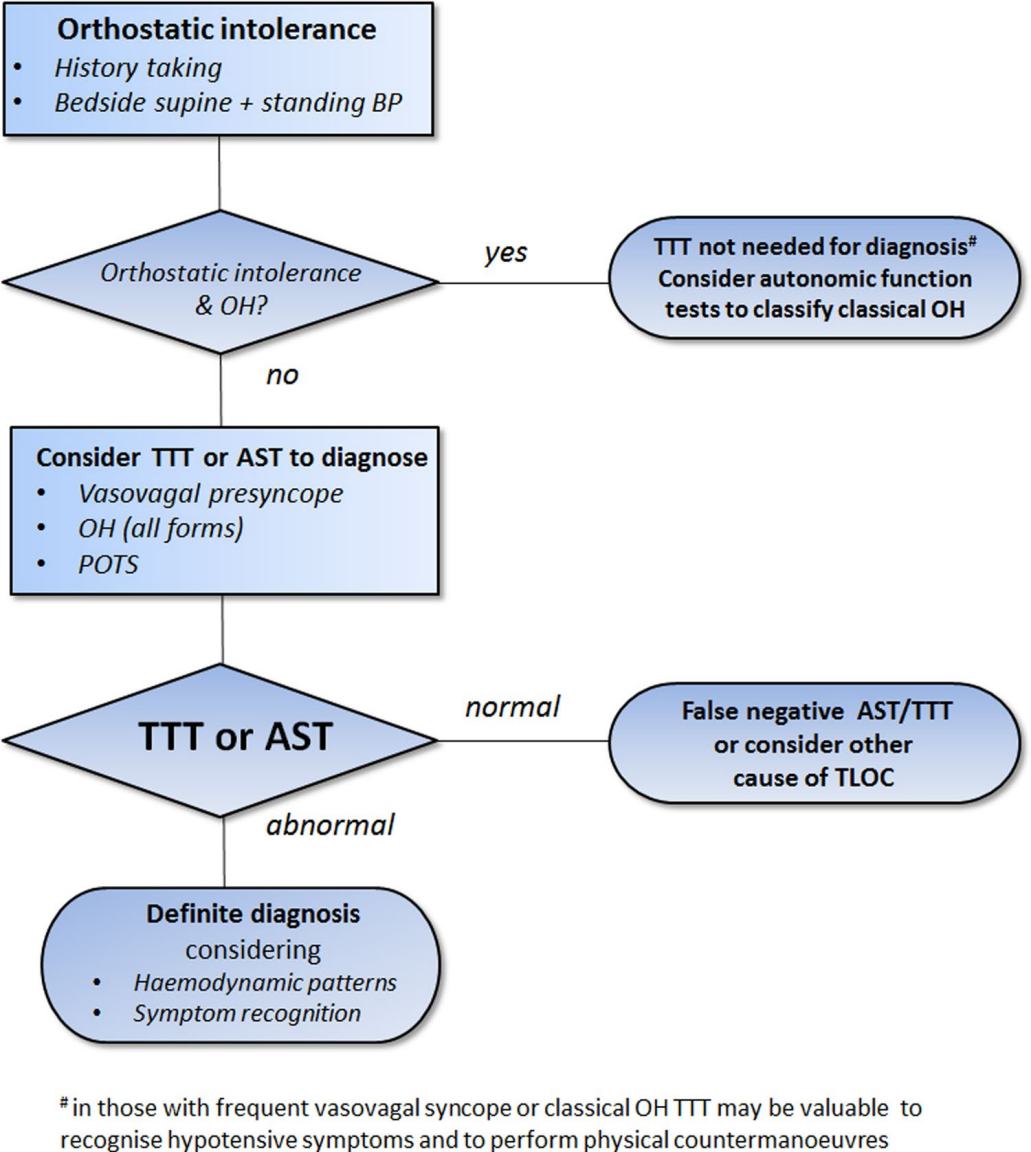
Flow chart of the diagnostic work-up of orthostatic intolerance, i.e. history taking and supine and standing blood pressure measurements. *AST* active standing test with continuous blood pressure measurements, *classic OH* classical orthostatic hypotension, *POTS* postural orthostatic tachycardia syndrome, *TTT* tilt table testing

TTT and other provocative cardiovascular autonomic tests primarily aim to obtain a pathophysiological correlate for orthostatic intolerance and TLOC. The fundamental tool for the differential diagnosis is history taking of patients and eyewitnesses [2, 4]. TTT may provide an important addition to history taking if the initial evaluation does not yield a definite or highly likely diagnosis. TTT should neither be used as a substitute for history taking nor isolated from history taking. Figure 1 shows a pragmatic approach, beginning with the initial evaluation of TLOC including history taking, ECG and bedside orthostatic blood pressure measurements. Figure 2 provides a flow chart for the work-up of patients with orthostatic intolerance.

### To obtain a pathophysiological correlate for orthostatic intolerance and TLOC

The primary aim of TTT is to provoke an event with complaint recognition and to demonstrate the pathophysiological correlate [2, 5]. These aspects are both crucial: recognition may concern subjective sensations reported by patients as well as visible aspects, such as changes in facial colour or movements, recognised by eyewitnesses as similar to spontaneous ones. Together with demonstration of pathophysiological measurements, a clinical-pathophysiological correlate is obtained, thereby proving the cause of TLOC. For most indications, TTT relies on prolonged orthostatic stress in the near-vertical position (‘head-up tilt’). TTT is not useful for all TLOC forms, as epileptic seizures and cardiac syncope are not commonly provoked by the upright posture. In contrast, TTT is useful for diagnosing syncope forms with an orthostatic component, i.e. reflex syncope and syncope due to orthostatic hypotension. TTT is also useful to provoke psychogenic TLOC, i.e. short-lasting apparent unconsciousness due to conversion. TTT can provide a definite diagnosis of psychogenic TLOC by excluding epilepsy or syncope. In the elderly it may be difficult to distinguish between falls with and without loss of consciousness. Hence, TTT may be used to investigate unexplained falls in the elderly, as these may be due to syncope [6, 7].

The role of TTT in diagnosing vasovagal syncope (VVS) has been debated [8–11]. The yield of a certain or highly likely diagnosis with history taking following an initial evaluation by hospital physicians and long-term follow-up as a reference may amount to 60%, with an accuracy of about 90% [12]. The diagnostic yield can increase to as much as 85% with additional history taking by an expert in syncope [13]. In view of these findings, TTT is not needed in the majority of cases presenting with syncope. Another concern is the specificity of TTT. Syncope during TTT reflects a tendency towards hypotension due to low preload in the upright position, rather than a specific diagnosis [14]. TTT modulates the occurrence not only of orthostatic VVS, but also of other forms of syncope such as cardiac syncope [14]. TTT should therefore only be performed after a careful and detailed medical history and examination. The results of the test should be interpreted in the context of that history and examination. Estimates of sensitivity (overall 59%; 95% CI 53–64%; range 21–72%) and specificity (overall 91%; 95% CI 87–93%) of TTT in diagnosing VVS differ [15]. Important factors contributing to the variability relate to the specific test protocol (e.g. the use of pharmacological provocation, tilt angle, duration of TTT, etc.), interpretation of the results, meaning whether complaint recognition or pathophysiological measurements are used as the standard to judge abnormality, and the lack of an agreed reference standard other than long-term follow-up [2, 5, 15, 16]. The most marked contrasts are seen between 'passive' TTT, i.e. upright tilt without pharmacological provocation [sensitivity 37%; (95% CI 29–46%); specificity 99% (95% CI 97–99%)], and ‘active’ protocols [e.g. nitroglycerin provocation sensitivity 66% (95% CI 60–72%); specificity 89% (95% CI 84–92%) [15]. As TTT relies predominantly on the orthostatic, literally meaning ‘upright’, position, TTT indications comprise the conditions bundled under ‘orthostatic intolerance’ [2, 5, 16]. Orthostatic intolerance includes all three forms of OH (i.e. initial, classic and delayed OH), delayed orthostatic BP recovery, orthostatic VVS and postural orthostatic tachycardia syndrome (POTS). Although some forms of reflex syncope, e.g. emotional VVS and cough syncope, primarily rely on other triggers, subjects with these forms are often also susceptible to orthostatic VVS [17, 18]. TTT may therefore still help to provoke syncope in these cases. In other cases, specific additional provocations may be needed to provoke TLOC.

The role of TTT and other provocative cardiovascular autonomic tests is similar for the three forms of orthostatic hypotension (OH), i.e. initial, classic and delayed, in that a definite diagnosis can be made when complaint recognition coincides with the type of BP decrease that is specific for each form (Table 1). The three forms may, however, require specific test protocols: (1) initial OH is commonly not provoked by passive tilt, but requires an active standing test with continuous BP measurements [19, 20]; (2) classic OH is most commonly screened at bedside with intermittent BP measurements, but can also be assessed with TTT or an active standing test [21]; delayed OH may require prolongation of the test protocol to allow a blood pressure decrease to occur [21–23]. Note that classic OH and presumably delayed OH often fluctuate in severity, so the absence of complaints and a blood pressure decrease during the test do not exclude OH (Table 2) [2, 21]. Conversely, the finding of classic OH does not automatically provide an explanation for orthostatic intolerance or TLOC, as it is a common finding affecting up to one in five community-dwelling elderly [2, 24]. When there is such a discrepancy between measurement results and complaints, the question of whether the measured OH represents a clinically relevant finding critically depends on the clinical presentation. When doubt remains, home measurements during complaints may settle the issue.

**Table 1:** Haemodynamic criteria for conditions causing orthostatic intolerance [3, 41]

TTT/AST indication	Fall in SBP upon standing	Fall in DBP upon standing	Increase in HR upon standing	Timing
Initial OH	> 40 mmHg	> 20 mmHg	Not specified	Transient BP fall within 15 s upon standing
Classic OH^a,b^	≥ 20 mmHg^f^	≥ 10 mmHg	Not specified^c^	Sustained BP fall within 3 min standing
Delayed OH^a^	≥ 20 mmHg	≥ 10 mmHg	Not specified	Sustained BP fall > 3 min standing
POTS^d^	SBP fall not meeting OH criteria	DBP fall not meeting OH criteria	> 30 bpm^e^ or > 120 bpm	Sustained HR increase within 10 min standing
Vasovagal presyncope	No formal criteria^g^	No formal criteria^g^	No formal criteria^g^	No formal criteria^g^
Delayed orthostatic BP recovery	Inability of SBP to recover to supine values within 30 s of standing. Standing SBP should be ≥ 20 mmHg lower than supine values but not meet criteria of classic or initial OH	Not meeting initial OH/classic OH criteria	Not specified	BP fall within 30 s upon standing

*AST*  active standing test, *classic OH*  classical orthostatic hypotension, *DBP*  diastolic blood pressure, *delayed OH* delayed orthostatic hypotension, *HR* heart rate, *initial OH* initial orthostatic hypotension, *POTS* postural orthostatic tachycardia syndrome, *SBP* systolic blood pressure, *TTT* tilt table test

^a^BP fall should be sustained to avoid confusion with transient BP falls seen in initial OH or VVS

^b^Please note that the ESC guidelines [2] added an additional (optional) criterion of SBP < 90 mmHg, as these values are strongly associated with the occurrence of symptoms. An abnormal orthostatic fall in DBP without an abnormal fall in SBP is rare among patients with syncope and orthostatic intolerance [86]

^c^A ΔHR/ΔSBP ratio < 0.5 bpm/mmHg argues for neurogenic OH [27]

^d^POTS is a clinical diagnosis and requires symptoms of orthostatic intolerance and the documentation of an exaggerated postural tachycardia using TTT or an active standing test

^e^For individuals aged 12–19 years, the required increment is > 40 bpm

^f^For patients with supine hypertension, a reduction in SBP ≥ 30 mmHg is required, as the magnitude of the orthostatic BP fall is dependent on the baseline BP

^g^No formal criteria to differentiate between vasovagal (pre)syncope and other causes of presyncope. The haemodynamic patterns associated with vasovagal (pre)syncope are heterogeneous [30] and may or may not be accompanied by HR slowing. Various criteria have been proposed to differentiate between subtypes

**Table 2:** Association of orthostatic intolerance and orthostatic hypotension [2] Reproduced with permission from the 2018 ESC Guidelines for the diagnosis and management of syncope

		History of syncope and orthostatic complaints	
		*Highly suggestive of OH *(pre)syncope present during standing, absent while lying, and less severe or absent while sitting; a predilection for the morning; sitting or lying down must help; complaints may get worse after exercise, after meals or in high temperatures; no signs of ‘autonomic activation’ (sweating, nausea, etc.)	*Possibly due to OH* not all of the features highly suggestive of OH are present
Supine and standing BP measurement	Symptomatic abnormal BP fall	Syncope is due to OH	Syncope is likely due to OH
	Asymptomatic abnormal BP fall	Syncope is likely due to OH	Syncope may be due to OH
	No abnormal BP drop	Unproven	Unproven

*BP* blood pressure, *OH* orthostatic hypotension

A diagnosis of psychogenic TLOC preferably requires documentation of an event, for which video or video-EEG recordings provide the most convincing evidence. History taking often raises a strong suspicion of psychogenic TLOC, but may not always be reliable enough to rule out other causes of TLOC [25]. The use of video-EEG is particularly important to exclude epilepsy when the clinical event is accompanied by positive motor phenomena [25]. Note that a normal EEG during a provoked event excludes syncope with certainty, but need not exclude all possible forms of epilepsy. It does, however, exclude those forms of epilepsy that present with TLOC, i.e. tonic, clonic and tonic–clonic seizures [26]. A diagnosis of psychogenic TLOC can also be established on clinical grounds if a clinician witnesses the event and observes features favouring psychogenic TLOC (e.g. eye closure, resisted eye-opening, partial responsiveness during the event, etc.) [25].

### To classify classic OH

TTT and other autonomic tests (active standing test, Valsalva manoeuvre and deep breathing) may help to distinguish between neurogenic and non-neurogenic causes for classic OH and to identify sympathetic or parasympathetic dysfunction in those with neurogenic OH [2, 21, 27, 28]. A blunted heart rate increase during classic OH makes a neurogenic cause more likely. A ΔHR/ΔSBP ratio < 0.5 bpm/mmHg argues for neurogenic OH (sensitivity 91%; specificity 88% AUC = 0.96) [27]. It should be noted, however, that cardiac disorders (e.g., pacemaker, arrhythmias, etc.) or certain drugs may preclude heart rate augmentation; thus an ECG, cardiac history and a medication review should be part of the evaluation of classic OH [21]. Specialised autonomic function tests including responses to deep breathing and Valsalva manoeuvre may also help to diagnose neurogenic OH as well as to identify sympathetic or parasympathetic dysfunction in those with neurogenic OH [2, 21, 28–31]. The absence of a blood pressure overshoot and the absence of a heart rate increase during the Valsalva manoeuvre is pathognomonic for neurogenic classic OH. There is also consensus that a blunted or abolished heart rate variation during deep breathing is suggestive of neurogenic classic OH. Interpretation of these findings requires age-adjusted, normative values [31].

### Treatment

TTT is not completely restricted to diagnosis. Patients may exhibit a decrease in syncope frequency after TTT, which may be due to patients having learned to recognise early signs of syncope, allowing them to take measures to prevent it [32]. TTT may easily be expanded with a biofeedback session to teach the ‘counter manoeuvres’ [33, 34]. This session can be performed when subjects are still symptomatic after syncope, thus allowing strong feedback regarding the effectiveness of these interventions. The session is also extremely important for patients’ education, as patients can see their own blood pressure fluctuate and discover which manoeuvres decrease (e.g., squat-to-stand) and which manoeuvres increase (e.g., leg crossing) their blood pressure. Applying these measures using biofeedback reduces the recurrence risk in patients with reflex syncope by 39% compared to conventional treatment only, i.e. explanation and life-style advice [33]. Patients with definite VVS and no need for additional testing could be referred to an animated video on physical counter manoeuvres [35]. TTT also allows the study of the temporal relation between onset of asystole and TLOC in those with asystolic VVS and helps to guide pacemaker recommendations. In one-third of cases of tilt-induced asystolic reflex syncope, asystole occurred too late to have been the primary cause of TLOC, thus making pacemaker implantation likely ineffective [36].

#### Recommendations

##### TTT should be considered

- To increase the probability of a diagnosis of reflex syncope
  This holds for those in whom the initial evaluation, including history taking, ECG, and supine and standing blood pressure measurements raised a suspicion but not a definite or highly likely (probability 80–100%) diagnosis, or in those in whom confirmation of a diagnosis is required for other purposes, such as convincing patients, parents or medicolegal requirements.
- To assess classic OH and delayed OH
  The recommended screening test for classic OH is a bedside active standing test with conventional intermittent BP measurements. TTT or an active standing test with continuous BP measurements should be considered if the bedside test does not show classic OH while the symptoms suggest classic OH (Table 2) [2]. TTT is particularly useful in detecting delayed OH, i.e. those with a sustained blood pressure fall of the magnitude of classic OH, but occurring later than 3 min upon standing [3, 22]. TTT may also be used to diagnose classic OH in subjects for whom active standing is difficult or unlikely to succeed, such as frail elderly and patients with significant motor impairments.
- To support a clinical diagnosis of POTS [3, 37]
  Documentation of an exaggerated postural tachycardia using TTT or an active standing test may support a clinical diagnosis of POTS.
- To differentiate between syncope with myoclonus (‘convulsive’ syncope) and tonic–clonic seizures [38].
- To discriminate between neurogenic and non-neurogenic classic OH
  A blunted heart rate increase during classic OH makes a neurogenic cause more likely. A ΔHR/ΔSBP ratio < 0.5 bpm/mmHg argues for neurogenic OH [27].
- To differentiate between vasovagal syncope and psychogenic TLOC [39, 40].
- To study the timing between asystole and the onset of TLOC
  Asystole in VVS may occur too late to have been the prime cause of TLOC, making pacemaker implantation likely ineffective [36].
- To teach patients with reflex syncope and orthostatic hypotension to recognise hypotensive symptoms and how to perform physical counter manoeuvres [33, 34].

Counter manoeuvres are effective measures to counteract impending reflex syncope or syncope due to orthostatic hypotension. Applying these measures using biofeedback reduces recurrence risk in patients with reflex syncope by 39% compared to conventional treatment (i.e. explanation and lifestyle advice) [33].

##### TTT should not be used

- To evaluate the treatment of reflex syncope
  TTT should not be used for the routine clinical evaluation of treatment efficacy, although it might have a role in the evaluation of treatment approaches for reflex syncope in the research context [2, 11].
- To exclude cardiac syncope
  Syncope during TTT suggests the presence of hypotensive susceptibility. This tendency is common and modulates the occurrence not only of orthostatic VVS, but also of other forms of syncope such as cardiac syncope [14].
- To exclude VVS
  The sensitivity of TTT, especially TTT without pharmacological provocation, is too low to exclude VVS [14, 15].
- To diagnose initial OH

Documentation of initial OH requires an active standing test with a beat-to-beat blood pressure monitor (see paragraph 5.2) [19, 20, 41].

## Indications for additional provocations and measurements

**Fig. 3:**
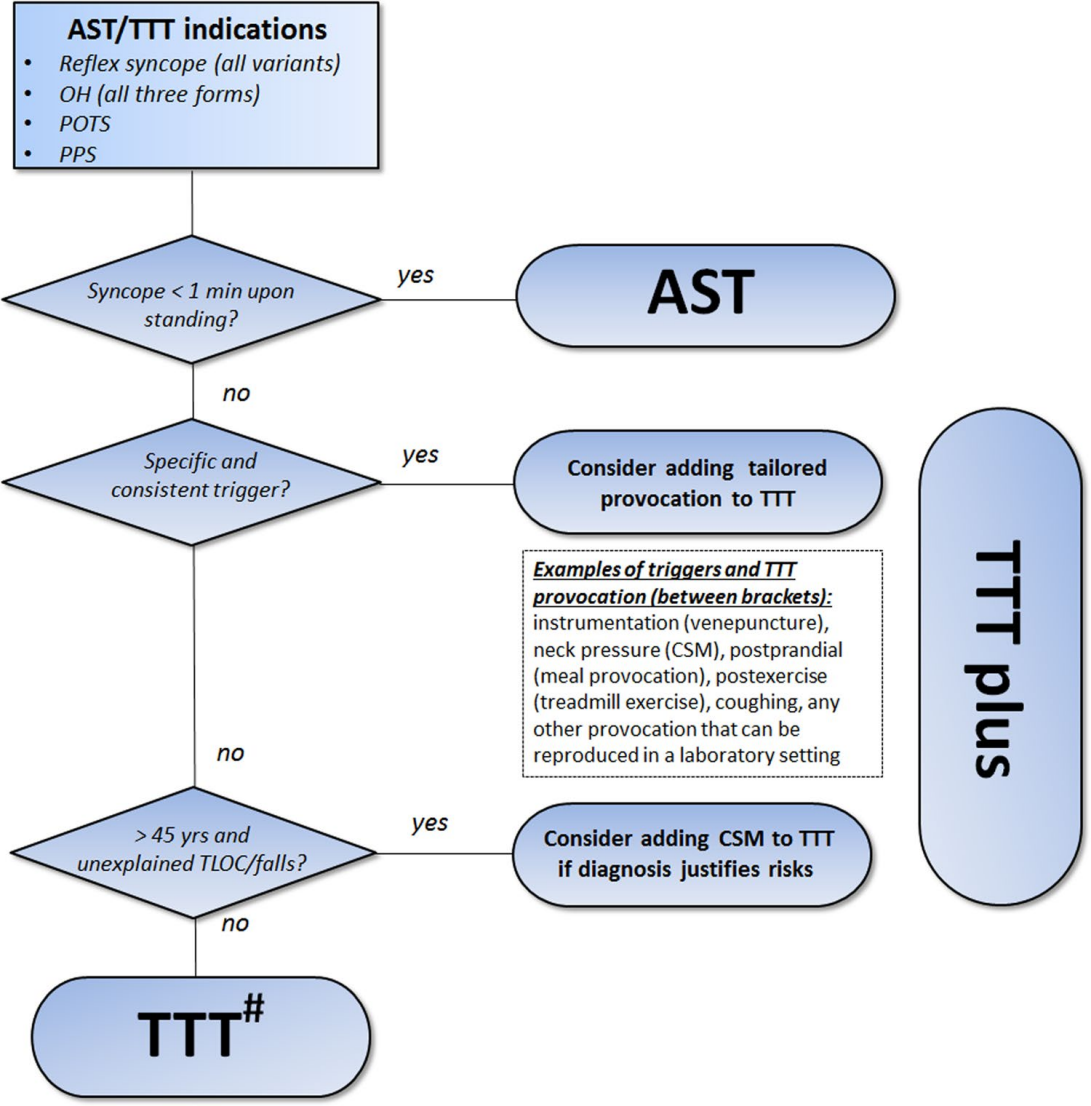
Choice of type of tilt table testing (TTT) protocol. ^#^Pharmacological provocation with sublingual trinitroglycerin (TNG) may be considered to increase the sensitivity for VVS but should be avoided in those for whom delayed OH is suspected. *AST* active standing test with continuous blood pressure measurements, *CSM* carotid sinus massage, *OH* orthostatic hypotension, *POTS* postural orthostatic tachycardia syndrome, *PPS* psychogenic pseudosyncope, *TTT* tilt table testing

The committee recommends that additional provocations and measurements are tailored to the patient's needs. Figure 3 provides guidance as to when these auxiliary measures could be considered. Recommendations for TTT protocols and supplementary measurements are summarised in Table 3 and detailed in the following section.

**Table 3:** TTT protocols and supplementary measurements for each TTT indication

TTT indication	Duration of tilt^a^	Pharmacological provocation	Additional measurements
Orthostatic intolerance^b^ - Classic OH - POTS - Delayed OH - Vasovagal presyncope	At least 10 min - 3 min - 10 min - Up to 40 min - Up to 45 min	Not recommended	*Optional* Video, EEG, Respiratory, TCD, Catecholamines
Transient loss of consciousness - Reflex syncope - PPS	Up to 45 min^c^	Optional	*Optional* Video, EEG, Respiratory, NIRS, TCD

*Classic OH*  classical orthostatic hypotension, *delayed OH* delayed orthostatic hypotension, *LBNP* lower body negative pressure, *NIRS* near-infrared spectroscopy, *POTS* postural orthostatic tachycardia syndrome, *PPS* psychogenic pseudosyncope, *TCD* transcranial Doppler

^a^TTT should be terminated earlier in the event a clinical endpoint (i.e. (pre)syncope) is reached

^b^Please note that an active standing test may also be used to establish a diagnosis in patients with orthostatic intolerance (see ‘Indications’ in the main manuscript)

^c^In the case of pharmacological provocation (TNG), TTT may be shortened to 40 min (20 min before and 20 min after TNG administration)

## Equipment

### Basic equipment

#### Beat-to-beat blood pressure monitor

In the context of syncope, blood pressure (BP) can change substantially in a few seconds, so it must be measured with an appropriate temporal resolution. Conventional intermittent BP measurements allow one measurement per minute at best and are therefore unsuitable for syncope assessment. Classic OH and delayed OH are, however, accompanied by sustained decrease in BP lasting minutes, and hence can be detected with conventional intermittent BP measurements [19, 21, 42]. Delayed OH, however, often occurs after 10 min of passive standing [22]. TTT is therefore more appropriate for identifying delayed OH. In frail elderly and those with major motor impairments, TTT may also be preferred over active standing to prevent falling. Several commercial devices are available for non-invasive beat-to-beat measurements of blood pressure and heart rate using finger photoplethysmography and the volume clamp method [43]. Additional software yields estimates of other circulatory parameters including stroke volume, cardiac output and total peripheral resistance; these may be used to quantify the relative contributions of vasodilation and cardioinhibition [44], but this is not required for routine clinical testing.

#### ECG

At least one ECG channel is required to detect bradycardia or asystole and to identify its underlying mechanism (e.g. intermittent AV block) [45, 46].

#### Tilt table

The tilt-down time should preferably be short for syncope, as longer times may lengthen circulatory arrest and thus the duration of cerebral hypoperfusion [47]. The optimal time is not known. The panel recommends the tilt-back period from 70° to horizontal should be less than 15 s. The subject should be protected against falling with safety straps, with at least one strap at the chest and one above the knee.

### Equipment extensions

#### Video and EEG recordings

For reflex syncope, adding video to TTT has the advantage that key clinical events that usually last less than 20 s can be studied in detail after the fact. Adding EEG provides additional guidance in control over the tilt procedure in that slowing of the EEG should always result in tilting the patient back; in reverse, tilting back is not yet needed if the EEG is still normal. The EEG pattern consists of either slowing or a slow-flat-slow pattern; this provides information about the degree of cerebral perfusion [38, 48]. Adding video and EEG has limited clinical value in OH because TTT in OH forms rarely induces syncope, and if complaints occur, these usually concern presyncope only, not usually accompanied by EEG changes.

For psychogenic TLOC, video and EEG recordings during TTT are very important, as they enable a definite diagnosis of psychogenic TLOC [2, 39, 40] and recognition of mixed presentations of psychogenic pseudosyncope and VVS [40]. A complete clinical identification of the presence of TLOC requires assessment of loss of responsiveness, abnormal motor control, amnesia for the period of apparent unconsciousness and a short duration [2, 26]. Adding video allows these phenomena to be assessed with certainty. The addition of EEG helps to confirm the presence of normal brain activity during psychogenic TLOC and thereby to exclude TLOC due to syncope (slowing and/or flattening) or epilepsy (epileptiform abnormalities). Video without EEG does allow a highly probable diagnosis of psychogenic pseudosyncope, i.e. attacks with loss of muscle tone but without jerking movements, by proving that the recorded event fulfils the clinical criteria of TLOC in the absence of hypotension [25, 39, 40]. If TLOC is accompanied by stiffening or jerks, however, additional EEG recordings are recommended to discriminate between psychogenic non-epileptic seizures and epileptic seizures [25]. A diagnosis of psychogenic TLOC can also be established on clinical grounds if a clinician witness observed TLOC and documented examination findings typically found in psychogenic TLOC (e.g. eye closure, resisted eye-opening, partial responsiveness during the event, etc.) [25].

Adding video-EEG to TTT can be accomplished by using an EEG machine to record and store all signals, including the output from the beat-to-beat BP monitor. As this is easily accomplished in a neurological setting, the panel encourages adding video and EEG to most TTT procedures. The video should record facial expression to assess eye closure and skin colour and, preferably, the upper extremities to evaluate the presence of jerking movements.

#### Respiratory recordings

Respiration can be recorded using strain gauges around the thorax or abdomen, blood oxygen saturation and end-tidal CO_2_. Of these, CO_2_ recordings may offer the most insight. CO_2_ recordings are useful to detail the pathophysiological cascade, as hypocapnia may precede reflex syncope [49]. Adding CO_2_ recordings to TTT may also be of clinical interest, as it may help to identify POTS subtypes that are consequent upon postural hyperventilation [50] or episodes of hyperventilation in those who report dizziness without concomitant hypotension [51].

#### Blood sampling

Blood sampling of neuro-endocrine substances holds promise as a means to improve pathophysiological understanding [21, 52–54]. Measuring catecholamines before and after head-up tilt offers insights into baroreflex-mediated sympathetic activation, mostly to help distinguish between causes of neurogenic OH such as pure autonomic failure, Parkinson's disease and multiple system atrophy, and to classify POTS variants [21, 52–55]. Catecholamine assessments may also help to predict conversion from pure autonomic failure to multiple system atrophy [56]. The measurements require placement of an indwelling venous line and established protocols regarding the material (cooling, centrifugation and type of assay) and TTT, as the catheter implies a needle to obtain venous or arterial access and may thereby provoke VVS. To avoid this, a longer period of supine rest is recommended before tilt up. The panel recommends restricting the use of catecholamine assays to those with a diagnosis of neurogenic OH where the clinical work-up fails to discriminate between pre- and postganglionic causes.

#### Transcranial Doppler (TCD)

TCD monitoring during TTT aids in the assessment of alterations in cerebral blood flow velocity at a high temporal resolution. This is of interest for investigating the pathophysiology of syncope [57], but has so far been of no help in the differential diagnosis of orthostatic intolerance or TLOC. TCD monitoring has been advocated as an additional tool to help establish psychogenic pseudosyncope [58].

#### Near-infrared spectroscopy (NIRS)

Near-infrared spectroscopy of the brain reflects the relative amount of deoxygenated blood in the scalp and brain. At present, the technique shows promise. as it suggests that decreased brain perfusion may be identified prior to clinical manifestations [59, 60].

## TTT protocols

### Classic TTT

We first present classic TTT and then discuss all variants and extensions that can be performed using the same equipment. The choice of the TTT protocol will depend on the clinical presentation (Figs. 2, 3).

#### Indication reflex syncope

Several protocols for TTT have been reported with or without pharmacological challenges [15, 61]. The available evidence suggests that 30–60 min is optimal for the diagnosis of VVS [2, 5, 15]. The panel did not reach consensus as to whether TTT should be performed with or without pharmacological provocation. While the majority of the panel preferred to perform TTT for 45 min without pharmacological provocation, there is evidence that pharmacological provocation increases sensitivity, with a minimal effect on the number of false positive results [15, 62]. For pharmacological provocation, we advocate against the use of intravenous isoproterenol and instead recommend sublingual trinitroglycerin (TNG) (300–400 mcg): TNG does not require cannulation and is easier and quicker to administer, while sensitivity and specificity of TNG and isoproterenol are similar [15]. TNG is usually administered in a fixed dose (0.3 or 0.4 mg) rather than a dose based on body mass. It is not known at which mass side effects become a limiting factor, but the panel discourages the use TNG in those with a mass below 50 kg. Arguments favouring TNG provocation include the increased sensitivity for VVS [sensitivity 66% (95% CI 60–72%) vs passive TTT (37%; (95% CI 29–46%)] [15]. TNG may also impact specificity, albeit to a lesser extent. The lower specificity of the TNG protocols [89% (95% CI 84–92%) vs passive 99% (95% CI 97–99%)] requires a critical evaluation of whether the provoked event resembles the spontaneous one. Another drawback of TNG provocation is that it may provoke delayed OH. This is of particular concern in those with orthostatic (pre)syncope without specific clues for VVS or delayed OH (e.g. orthostatic (pre)syncope without autonomic activation in an elderly subject). Another factor to consider is the health care setting, as in some countries application of TNG for diagnostic purposes may be restricted or require a specific clinical environment.

Lower body negative pressure (LBNP) is an alternative non-pharmacological measure to provoke syncope during TTT. While LBNP has been studied extensively to assess haemodynamic responses to orthostatic stress, little is known of the validity of LBNP to detect VVS in patients with unexplained TLOC [63, 64]. This does not mean that LBNP cannot be useful in this context, but its clinical value has not yet been proven.

- Supine rest phase of at least 5 min with reliable and constant BP and HR values as a baseline.
- Head-up tilt angle between 60° and 80°, measured from the horizontal [15]. At 60°, 70° and 80°, the force component pulling blood towards nether parts of the body presents 0.87, 0.94 and 0.98 of that of the fully vertical position. The positive yield of TTT seems optimal at 70°, as the sensitivity is higher compared with TTT at 60° and 80° [15].
- TTT without pharmacological provocation: head-up tilt for 45 min,
- TTT with pharmacological provocation: start with 20 min head-up tilt, followed by administration of TNG in the tilted position and continuation for another 20 min,
- TTT can be terminated when clinical events occur or, in case no symptoms are provoked, when the end of the recording period is reached. Which symptoms can serve as an endpoint will critically depend on the clinical context. Presyncope may be used as an endpoint in cases with a high pre-test likelihood of reflex syncope; patients may then be tilted back when recognised complaints are accompanied by hypotension. When the a priori probability is low, or when syncope with myoclonus needs to be distinguished from a convulsive seizure, syncope should be the endpoint. The decision to tilt back should be taken by someone with direct access to the patient and all recorded signals. The presence of asystole or EEG slowing, if recorded, is always a reason to tilt back immediately.
- A key element of TTT is to ask patients for recognition of symptoms. Eyewitnesses may confirm whether the provoked event resembled the spontaneous episodes, which may help to reduce false positive results.

#### Indication OH

Pharmacological provocation should not be used for any form of OH. Initial OH is not a reason for a TTT, but requires an active standing test with continuous blood pressure measurements. TTT may be used for those with orthostatic intolerance (1) when the active standing test does not reveal classic OH, (2) when delayed OH is suspected at history taking or (3) when a complaint correlation is needed. The TTT duration may be tailored to individual needs. Syncope occurs rarely in classic or delayed OH during TTT and should not be the primary endpoint.

- Supine rest phase of at least 5 min before head-up tilt, with reliable and constant BP and HR values as a baseline. Supine measurements can be used to assess neurogenic supine hypertension [65].
- Head-up tilt angle between 60° and 80°, measured from the horizontal.
- Head-up tilt of 3 min would suffice to establish a diagnosis of classic OH, but longer TTT may be needed to rule out other causes of OI (e.g. delayed OH, POTS). The diagnosis of delayed OH may require a head-up tilt of up to 45 min [22, 23]. Patients may be tilted back when a complaint correlation is obtained, when syncope occurs or when patients can no longer withstand the head-up position.
- Complaint recognition: see above. It is important to note that a mean standing BP < 75 mmHg had optimal sensitivity (97%) and specificity (98%) for detecting symptomatic OH in patients with Parkinson’s disease [66]. The symptom threshold may, however, vary between causes of OH. In a study of patients with diverse causes of OH, many patients were asymptomatic despite a substantial fall in SBP and low orthostatic blood pressure, suggesting that symptoms may not always be a reliable indicator of impaired cerebral perfusion or risk of syncope [67].

#### Indication psychogenic TLOC

For psychogenic TLOC, pharmacological provocation may be used as for reflex syncope, for two reasons: firstly, VVS occurs more often in psychogenic pseudosyncope than chance predicts [40] and the occurrence of psychogenic TLOC may depend on suggestion [68], which may be aided by a provocation procedure. The same protocol may be used for psychogenic TLOC as for reflex syncope, except for the endpoint.

- Ensure a supine rest phase of at least 5 min before head-up tilt.
- Head-up tilt angle between 60° and 80°, measured from the horizontal.
- Head-up tilt for up to 40 min (pharmacological provocation) or 45 min (no pharmacological provocation).
- Pharmacological provocation may be chosen and performed as for reflex syncope.
- The test ends when the allotted time has passed or when psychogenic TLOC occurs. As blood pressure is not low and cerebral hypoperfusion is absent in psychogenic TLOC, tilting back may safely be postponed until all symptoms and signs are recognised.
- Complaint recognition: see above.

#### Indication POTS

Documentation of an exaggerated postural tachycardia using TTT or an active standing test may support a clinical diagnosis of POTS. The panel recommends the use of TTT in those with complaints of both orthostatic intolerance and TLOC, as TTT has the advantage of provoking VVS as well. The diagnostic criteria of POTS require a head-up tilt period of 10 min. If other causes of orthostatic intolerance are considered (notably vasovagal presyncope or delayed OH), a longer head-up tilt is needed to establish a diagnosis. As the pathophysiological relation between orthostatic tachycardia and symptoms is as yet unclear, the panel stresses that orthostatic tachycardia without symptoms is a non-specific finding. Dehydration should be considered as an explanation. The test does not aim to provoke syncope, but if it occurs, it should be considered that the initial high heart rate upon standing may be a reflection of compensatory mechanisms and thus an expression of VVS. In such case it would be worthwhile repeating TTT to see whether POTS is a consistent finding.

- Ensure a supine rest phase of at least 5 min before head-up tilt, with reliable and constant BP and HR values as a baseline.
- Head-up tilt angle between 60° and 80°, measured from the horizontal.
- Head-up tilt for 10 min.
- The test ends when the allotted time has passed, when 10 min have passed or when syncope inadvertently occurs, in which case the rules for tilting back of reflex syncope apply.
- Complaint recognition: see above.

### TTT: possible extensions and variants

#### Active standing test with continuous blood pressure measurements

The active standing test can be used to provoke initial orthostatic hypotension (initial OH), classical OH (classic OH) or POTS, or to demonstrate delayed orthostatic BP recovery. It includes supine rest for at least 5 min followed by 3 min of standing. For a more detailed description of the active standing test, see the recent review by Finucane et al. [19]. A diagnosis of initial OH can be established if a transient BP drop (> 40 mmHg systolic blood pressure or > 20 mmHg diastolic blood pressure) occurs within 15 s of standing. [3] Delayed orthostatic BP recovery is defined as the inability of systolic BP to recover to supine baseline values within 30 s of standing; the orthostatic BP should be ≥ 20 mmHg lower than the supine baseline values, but should not meet the criteria of classic or initial OH [41]. In view of the short duration of the blood pressure decrease in initial OH, the active standing test requires a beat-to-beat blood pressure measurement device. The active standing test can also be used to detect classic OH, as the criteria only require a measurement period of 3 min after standing up. In contrast to initial OH, classic OH or delayed OH can be detected with conventional intermittent BP measurements [42].

#### Valsalva manoeuvre and deep breathing

Autonomic function tests may help to (1) identify autonomic failure as the underlying cause of classic OH (2) to characterize cardiovascular sympathetic and parasympathetic autonomic function and (3) quantify the severity of autonomic dysfunction [2, 28, 30, 31]. To ensure valid results, it is extremely important that the tests are performed by trained personnel and under controlled circumstances, meaning a quiet and temperature-controlled room; no meals for 3 h before the test, and no vasoactive substances (nicotine or caffeine-, theine-, or taurine-containing drinks) or medications on the day of examination. During the Valsalva manoeuvre, the patient is asked to conduct a maximally forced expiration for 15 s with an open glottis, i.e. with open nose and mouth, or into a closed loop system with a resistance of 40 mmHg. During the deep breathing test, the patient is asked to breathe deeply at six breaths per minute for 1 min.

#### Carotid sinus massage (CSM)

The diagnostic yield of the TTT can be enhanced by adding carotid sinus massage (CSM). CSM has been advocated in adults over 40 years of age with a history of unexplained reflex syncope or falls, and those in whom TLOC is triggered by head rotation or pressure on carotid sinus [2, 69]. The need for a definite diagnosis should always be weighed against the risk for complications (see “Safety”).

#### Venepuncture

Adding venepuncture provocation may increase diagnostic yield, especially in those with a history of syncope during instrumentation [17]. If intended for blood sampling during the test it is recommended to be performed at least 20 min before TTT, so as to avoid provocation.

#### Meal provocation

A meal provocation can be used to diagnose postprandial hypotension [70]. Postprandial hypotension can be detected with conventional intermittent BP measurements. The panel recognises that there is no consensus regarding the definition and the assessment of postprandial hypotension. Most authors define postprandial hypotension as a fall of ≥ 20 mmHg in systolic blood pressure or SBP drop from > 100 mmHg to SBP < 90 mmHg within 2 h after completion of the meal [70]. Various provocations have been used including orally administered glucose, a standard (normal) meal or a mixed liquid meal [70]. Consensus is also lacking regarding the frequency of measurements and the body position during the test (supine, sitting) [70]. The panel recognises the need for uniform definitions and protocols to foster our understanding of postprandial hypotension.

#### Tailored provocations

A personally tailored provocation may be carried out in those with a specific and consistent trigger of TLOC. Examples include standing still following exercise [71, 72], coughing [18] or bending forward [45]. It should, however, be noted that many specific triggers suggest situational reflex syncope with high confidence and usually do not require diagnostic confirmation. Tailored provocation should therefore be reserved for those with unusual triggers or specific triggers but conflicting ictal signs or symptoms. Some provocation may require precautions to avoid injuries, such as floor mats.

#### Counter-pressure manoeuvres

The beat-to-beat blood pressure monitor can be used to teach patients suffering from reflex syncope or orthostatic hypotension to recognise hypotensive symptoms and to perform physical counter manoeuvres [33, 34]. Various manoeuvres including leg crossing, handgrip or arm tensing can be taught while patients view the blood pressure response on the monitor [33, 34]. Such training sessions allow tailoring of the manoeuvres. For example, leg crossing may not be feasible for those with movement disorders, but handgrip, arm tensing or tensing of the buttocks may help to improve orthostatic tolerance. Ideally, these manoeuvres are taught when patients experience mild symptoms of OH without the risk of immediate syncope or falls, e.g. immediately following TTT or active standing test.

## Patient information

The panel recognises that there is ample literature to support specific patient recommendations to prepare for TTT. It could be considered to instruct patients to refrain from easily preventable vasoactive substances (e.g. caffeinated beverages, tobacco smoking) as this may limit the ability of TTT to provoke symptoms. The recommendation regarding medication fully depends upon the clinical context: if symptoms are likely provoked by medication, the patient should continue the drug regimen. In other cases, it may be necessary to test the patients off medication, as certain drugs may confound the assessment of autonomic functions. In either case, reporting of the drug regimen is important to properly interpret TTT, as drug-induced orthostatic intolerance is a major cause of all three patterns of OH, particularly among the elderly [2, 24]. The panel recommends advising patients to empty the bladder prior to test to avoid sympathetic stress [73] and incontinence in case TTT provokes syncope. Another consideration is the timing of the tests, as TTT is more likely to provoke syncope in the morning hours [74].

## Safety

TTT is safe, and major complications are very rare [2, 11, 75, 76]. Although we found no evidence that TTT is harmful, the low blood pressure during syncope might theoretically harm those with ischaemic disorders of the heart or brain, so risks and benefits should be weighed in such patients. There is an association between frequent syncope, defined as five or more syncopal spells during life, with more white matter lesions [77]. The panel felt that the advantages of a definite diagnosis, with possibly fewer future syncopal spells as a result, outweigh the theoretical risk of adding white matter lesions by one syncopal spell during TTT. TTT may provoke atrial fibrillation, but this is usually self-limiting [2]. Other side effects include headache or migraine following TNG challenge.

Complications following CSM are uncommon (< 0.5%) and predominantly involve transient neurological symptoms, while some cases with stroke have been reported [78–83]. One death after CSM resulting from massive brain infarction has, however, been reported [84]. The risks and benefits of CSM therefore have to be carefully weighed. According to the ESC guidelines, CSM should be undertaken with caution in patients with previous stroke, TIA or known carotid stenosis > 70% [2]*.* The American Heart Association (AHA) and the American College of Cardiology (ACC) guidelines discourage the use of CSM in patients with a carotid bruit, TIA, stroke or myocardial infarction in the preceding months, except if carotid Doppler excludes a significant stenosis [11]. The expert group noticed that the more stringent criteria by the AHA/ACC more closely resemble the selection criteria employed in the studies assessing complication risks (bruits: 3 out of 4 studies; myocardial infarction: 2 out of 4 studies). The expert group feels that the need for a definite diagnosis of carotid sinus hypersensitivity should always be weighed against the risk for complications. Ultrasound investigation of the carotid arteries may be carried out to screen for atherosclerosis. There is, however, no evidence that this approach prevents complications.

## Clinical environment

It is strongly recommended that TTT is an integral part of a clinical facility for the diagnosis and management of syncope and related symptoms, with dedicated staff and access to appropriate diagnostics and therapies. A consensus statement of the European Heart Rhythm Association (EHRA) offers guidance on how to set up such a facility [85]. The panel endorses the ESC recommendations regarding TTT performance: the test should be conducted by a physician, technician or a nurse trained in syncope and resuscitation [2]. Clinical TTT should typically be conducted within a clinical environment with established procedures for life-threatening events [85].
